# Relationship Between Paternalistic Leadership and Employee Innovation: A Meta-Analysis Among Chinese Samples

**DOI:** 10.3389/fpsyg.2022.920006

**Published:** 2022-07-01

**Authors:** Lin Lu, Kaiji Zhou, Yingzhao Wang, Sishi Zhu

**Affiliations:** ^1^School of Business Administration, Southwestern University of Finance and Economics, Chengdu, China; ^2^Department of Applied Social Sciences, The Hong Kong Polytechnic University, Hong Kong, Hong Kong SAR, China; ^3^Xiao Ping Executive Leadership Academy, Guangan, China; ^4^Qingtaishan Middle School Attached to Sichuan Normal University, Chengdu, China

**Keywords:** paternalistic leadership, meta-analysis, innovation, Chinese sample, moderation effect

## Abstract

The meta-analysis was conducted to examine the relationships between three dimensions of paternalistic leadership and employee innovation in Chinese enterprises. There exists over a decade of empirical research on the influence of paternalistic leadership on employee innovation in China, but the findings from the various studies are not consistent. Sixty-nine studies from 2009 to 2021 were included in the meta-analysis, and 154 effect sizes were examined. The study found that two dimensions of paternalistic leadership (benevolent leadership *r* = 0.396 and moral leadership *r* = 0.329) were positively associated with employee innovation. In contrast, the dimension of authoritarian leadership was negatively associated with innovation (*r* = −0.151). Moderator analyses found that gender, the education level of employees, time, and the type of evaluation served as meaningful moderators. The moderating effects of outcome measure, the type of data collection method, and the type of publication were not significant. We discuss our limitations, implications for future studies, and practical implications for organizational management.

## Introduction

Paternalistic leadership, a widespread and deep-rooted leadership style in oriental organizations, is one of the most widely cited factors of influence on employees' behaviors in the Chinese context. Amidst the increasing interest of enterprises and researchers in innovation, whether paternalistic leadership promotes or impedes employees' innovation in China has raised broad concern (Wang and Cheng, 2010; Cheng, 2020; Nazir et al., 2021). The body of research on the subject is extensive, but discrepancies in the findings mark the literature. Authors such as Jin et al. (2016), Wang Y. W. et al. (2021), and Xia et al. (2021) concluded that one of the dimensions of paternalism (authoritarian leadership, AL) was positively associated with employee innovation, while other researchers (e.g., Du and Wang, 2020; Liang, 2020; Li and Wang, 2021) found a negative correlation between them. Wu (2018) and Cheng (2020) found strong positive relationships between the two other dimensions of paternalism (benevolent leadership, BL and moral leadership, ML) and employee innovation. However, the results of other studies (Feng, 2009; Li et al., 2014; Chen and Hou, 2016; Wang, 2019) found that benevolent and moral leadership were weakly or even negatively correlated with innovation.

Empirical studies may reach different conclusions due to sampling or other factors (Hunter and Schmidt, 2004). Meta-analysis can correct statistical errors based on large samples and reach a more general conclusion. Therefore, it is necessary to conduct a meta-analysis of the relationship between paternalistic leadership and Chinese employee innovation. A meta-analysis already involves the relationship between paternalistic leadership and employees' innovation (Hiller et al., 2019). However, (1) it mixed Chinese samples with the samples from other countries and did not report the results based on Chinese samples; (2) the inclusion of Chinese samples was not comprehensive enough (i.e., the included Chinese samples were only retrieved from Chinese Social Science Citation Index, while many studies from other databases were neglected); (3) it only involved innovation as one of the indicators of employee performance, and there was no specific and detailed investigation into the relationship between paternalistic leadership and employees' innovation.

Based on the reasons mentioned above, to clarify the relationship between paternalistic leadership and employee innovation in the Chinese context, a meta-analysis exclusively focusing on Chinese employees' innovation (not overall performance) is needed. Drawing upon the insights from the employee creativity formation mechanism model (Wang et al., 2010) and self-determination theory (Deci and Ryan, 1985), we examine the associations of the three aspects of paternalistic (BL, ML, and AL) with Chinese employees' innovation. And we explore the potential moderators in the associations, including methodological moderators (e.g., type of innovation evaluation), demographical moderators (e.g., employee gender and education level), and a macro moderator (i.e., era background). Our findings provide evidence from China on how paternalistic leaders promote or impede employee innovation. Our limitations, theoretical implications, and practical implications are discussed.

## Literature Review and Hypotheses

### Paternalistic Leadership

To a large extent, paternalistic leadership reflects Chinese Confucian culture and family values. In traditional Confucian culture, the patrilineal family is the primary institutional unit of society. According to Confucianism's notion of the five relationships that form the basis of society, the father–son relationship is second only to the monarch–subject relationship. The father is considered the family's core and has absolute authority. The Chinese generalize the experience learned from the family to other organizations. A paternalistic leader of an organization tends to play a role similar to a father in the patrilineal family, and the subordinates play the role of “offspring.” The leader must have the majesty of a father, while the subordinates must have “son-like” loyalty and obedience (Farh and Cheng, 2000). In addition, Confucian culture places great emphasis on personal morality. And people often place higher moral expectations on those with higher social status. In the Confucian context, leaders must have a high moral quality. Otherwise, they will not be genuinely venerated by their subordinates. Finally, paternalism also emphasizes the responsibility of the father to protect and care for the family members, which transformed into the benevolence of the superior to the inferior in enterprises and other organizations (Zhou and Long, 2005).

Paternalistic Leadership is a notion similar to a traditional Chinese “fatherly Leadership.” Paternalistic leaders not only show the image of a “strict father” who maintains strict discipline and rules but also show kindness and care to subordinates. At the same time, they also set a good moral example for subordinates (Farh and Cheng, 2000; Zhu et al., 2022). Paternalistic Leadership consists of three sub-leadership styles, benevolent leadership (BL), moral leadership (ML), and authoritarian leadership (AL). BL refers to the behaviors that involve long-term concerns and support for the followers' work, life, and welfare (Ren et al., 2021). ML involves the leader's virtue, self-discipline, and selflessness. AL emphasizes the leader's absolute authority and control over subordinates and requires subordinates to accept the assignment and obey the leader unconditionally (Farh and Cheng, 2000; Aycan, 2006; Pizzolitto et al., 2022). The three substyles may lead to different reactions and outcomes of subordinates. Benevolent leaders may be responded with the followers' gratitude and reciprocation, and ML may increase the followers' respect for and identification with the leader. However, on the other hand, AL can lead to subordinates' obedience to, dependency on, and fear of the leader (Cheng et al., 2004; Farh et al., 2006; Niu et al., 2009; Chen et al., 2014).

### Employee Innovation

Scott and Bruce (1994) defined innovation as the process by which individuals or teams generate new ideas and put them into practice to improve performance. Specifically, individual innovation is divided into three stages: (1) discovering problems and generating new ideas to solve problems, (2) examining the ideas and seeking support, and (3) putting the ideas into practice (Janssen, 2000).

At present, the measurement tools for employee innovation mainly include innovative behavior scales and creativity scales developed by Scott and Bruce (1994), Zhou and George (2001), Janssen (2000), and Criscuolo et al. (2014). To understand innovation among Chinese employees, researchers localized the adaptations of these scales (Lu and Zhang, 2007). They also conceptualize innovation as a process including idea generation, promotion, and execution. Based on the conceptualization and measurement of Chinese employee innovation (Kim et al., 2018; Lee et al., 2020a,b; Watts et al., 2020; Lin et al., 2022), this study takes both innovative behavior and creativity as the indicators of employee innovation. To ensure the rigor of our research, we examine whether the associations of the three aspects of paternalistic leadership with innovation vary over different measurement tools in moderator analyses.

### Relationship Between Paternalistic Leadership and Innovation

According to the employee creativity formation mechanism model (Wang et al., 2010), employees' intrinsic motivation at work plays a crucial role in the relationship between leadership and innovation. Scott and Bruce (1994) first suggested that intrinsic motivation is the core of innovation. Shin and Zhou (2003), Shalley et al. (2009), Wang et al. (2010), and Siyal et al. (2021) suggested that leadership affects employee innovation by influencing employees' intrinsic motivation.

Self-determination theory (SDT) believes that people generally have three basic psychological needs: autonomy, competence, and relatedness, which lie at the heart of individual intrinsic motivation. Autonomy refers to an individual's voluntary choice to engage in certain activities according to their inner will and desire. People with a satisfied need for autonomy tend to engage in behaviors with intrinsic interest and motivation. The need for competence refers to an individual to experience that they are capable of performing an activity. Relatedness is the individual's need to obtain the care and understanding of others in the external social environment to experience a sense of belonging to the group (Deci and Ryan, 1985, 2000). Benevolent leaders focus on mobilizing resources to support subordinates to complete tasks and achieve career development and care for subordinates' wellbeing (Wang and Cheng, 2010). Care from a benevolent leader can satisfy the relatedness needs of subordinates, enhance employees' intrinsic motivation at work, and promote their initiative at work, which may, as a result, promote employees' innovation. For example, employees with a higher intrinsic motivation at work are more likely to go the extra mile to solve problems creatively.

An enduring emphasis in innovation research has been on the influence of positive or negative leader behaviors, such as supportive (Madjar et al., 2002) or abusive (Aryee et al., 2007) leader behaviors. Leaders who create a supportive environment not only allow their subordinates to have the freedom to experiment with innovation (Amabile, 1988; Siyal et al., 2021) but also provide positive and constructive feedback at work and encourage employees to find and solve problems by themselves, which is conducive to improving employees' intrinsic motivation and promoting employees' innovation (George and Zhou, 2007; Su et al., 2022). Authoritarian leaders, on the other hand, closely monitor employees and require them to follow the rules and orders strictly. They do not allow employees to participate in decision-making. As a result, the lack of autonomy under AL will reduce employees' intrinsic motivation (George and Zhou, 2001; Zhou and George, 2003; Gu et al., 2020) and impede their creative thinking. In addition, authoritarian leaders rarely encourage or support followers to develop or realize their ideas, restricting employees' innovative behavior.

According to the analysis above, we propose Hypothesis 1 and 2:

*H1: Benevolent leadership is positively associated with Chinese employees' innovation*.
*H2: Authoritarian leadership is negatively related to Chinese employees' innovation*.

ML displays qualities such as honesty, integrity, equity, and selflessness, which can win the trust of subordinates (Brown and Treviño, 2006; Niu et al., 2009), and enhance subordinates' psychological safety. Because innovation usually takes risks, subordinates under a trusted leader will be more willing to propose and apply their ideas and challenge the status quo without the fear of unfair punishment. A high level of psychological safety will improve employees' “psychological freedom,” fostering their innovative activities (Walumbwa and Schaubroeck, 2009). Therefore, moral leadership may promote employee innovation by improving subordinates' trust in the leader and psychological safety. We propose Hypothesis 3:

*H3: Moral leadership is positively associated with Chinese employees' innovation*.

### Moderators of the Relationship Between Paternalistic Leadership and Employee Innovation

#### Gender

According to role congruity theory, female and male employees have different preferences for leadership (Eagly and Karau, 2002). Compared with male workers, females are more inclined to work under more humanistic and relationship-oriented supervision (Boatwright and Forrest, 2000). Benevolent leadership and moral leadership emphasize leaders' concern for the followers and moral example, which might have a greater positive impact on female workers' motivation and initiative by creating a caring atmosphere and fulfilling female workers' needs, and then further promoting females' performance, including innovation. In addition, females' relationship orientation might make it easier for females to settle when they are under authoritarian leadership and reduces the negative impact of authority on females' innovation. Gender role, on the other hand, demands males to be more competitive and more power-oriented (Eagly et al., 2000). This might lead to more dissatisfaction, conflicts, or even counter-productive behaviors among the male subordinates under authoritarian leadership because they are less willing to obey the authoritarian leader (Brandt and Henry, 2012; Liu et al., 2021), which might even further enhance the negative impact of authoritarian leadership on male workers' innovation. Thus, the percentage of female employees in the samples can probably enhance the positive effects of BL and ML on innovation and reduce the negative impact of AL on innovation. We develop Hypothesis 4:

*H4: The percentage of female respondents can positively moderate the relationships between the three aspects of paternalistic leadership and innovation*.

#### Education Level

It has been revealed that employees with higher education levels relatively value autonomy, respect, and emotional incentive more at work (Oldham and Cummings, 1996; Shalley et al., 2004). For employees with higher education levels, the role of leaders is no longer to give specific guidance to their work but to help them set goals and provide support (Shalley and Gilson, 2004). Benevolent leaders support and care for the work and life of subordinates, which can meet the emotional needs of educated workers. As a result, subordinates with higher education levels might engage in their work with a higher level of intrinsic motivation and initiative, which are exactly what innovation demands. We also expect that education level has a similar effect on the relationship between moral leadership and innovation.

On the contrary, authoritarian leadership that emphasizes strict control over subordinates and requires unconditional obedience might have a worse impact on educated workers' inner motivation than less-educated workers. Thus, we develop Hypotheses 5 and 6:

*H5: The percentage of employees with higher education levels (college diploma or above) positively moderates the associations of benevolent leadership and moral leadership with employee innovation*.
*H6: The percentage of employees with higher education levels (college diploma or above) negatively moderates the relationship between authoritarian leadership and employee innovation*.

#### Outcome Measure

Original studies included in this meta-analysis adopted different questionnaires or scales to assess employee innovation. Although some innovation scales focus more on innovative behavior and other scales are inclined to measure creativity, the difference among these innovation scales is tiny conceptually because behaviors are also used as the primary indicator in creativity assessment. That said, the different outcome measurement tools may still affect the robustness of our research results. Therefore, the outcome measure is examined as a potential moderator. Instead of putting forward a certain hypothesis, we examine an exploratory research question assessing if there is considerable variation in the effect sizes caused by the different innovation scales.

#### Type of Evaluation and Data Collection

To examine whether common method bias affected previous studies' results, we test the moderating effect of the evaluation method of innovation: supervisor-evaluation (leaders' rating of each subordinate's innovation) vs. self-evaluation (employees' self-reported innovation). And we also examine the difference between different data collection methods (cross-sectional and longitudinal). When the prediction variables and the outcome variables come from the same evaluator or the data is captured at the same time point, it often leads to common method bias and more significant coefficients (Podsakoff et al., 2003). Previous meta-analyses in other research areas have revealed this phenomenon (Lee et al., 2020a,b). We develop Hypothesis 7 and 8:

*H7: Supervisor-evaluation yields weaker associations between three aspects of paternalistic leadership and Chinese employees' innovation than self-evaluation*.
*H8: Longitudinal data yields weaker associations between three aspects of paternalistic leadership and Chinese employees' innovation than cross-sectional data*.

#### Time

Era background may moderate the relationship between paternalistic leadership and employee innovation in China. As China has been opening up to the outside world for a few decades (since 1979) and the economy has been developing rapidly, people's attitudes and values are also gradually changing, which may weaken the cultural soil of traditional ideas, including paternalism.

Since 2010, China's total GDP surpassed Japan's to become the world's second-largest economy. According to the theory of value change (Inkeles, 1969; Inglehart, 1997), people tend to emphasize freedom and self-expression in an advanced industrial society. The popularity of authoritarianism, which emphasizes absolute obedience, might be wearing off in Chinese society and organizations (Zheng et al., 2020). Therefore, authoritarian leadership might have a greater negative effect on employees' intrinsic motivation since it is getting less accepted by current employees and impedes employee innovation. Meanwhile, the effects of BL and ML on employee innovation might also be influenced by era background but in the opposite direction. We propose Hypothesis 9 and 10:

*H9: The year of publication positively moderates the relationship between benevolent leadership and moral leadership and Chinese employees' innovation*.
*H10: The year of publication negatively moderates the relationship between authoritarian leadership and Chinese employees' innovation*.

#### Type of Publication

Generally, studies with significant results are easier to get published, making a meta-analysis overestimate the real effect size between variables (Sterne et al., 2000). To avoid this bias, this meta-analysis includes not only journal articles but also theses, dissertations, and conference papers, assessing the difference in the results between published journal articles and other studies (unpublished studies).

### Summary

This meta-analysis aims to address the abovementioned questions about the associations between the three aspects of paternalistic leadership and employee innovation. We examine the strength and direction of the associations of BL, ML, and AL with employee innovation in China. We expect BL and ML to correlate positively with innovation, but we are still curious about the possible difference in the size of these two associations. And we expect AL to relate negatively to innovation. We examine whether the size of the associations depends on sample features (the percentage of female employees and the percentage of employees with a college diploma or above) and methodological features (outcome measure, the type of evaluation, and the type of data collection method). We also test whether the strength and directions of the associations change over time (through the year of publication). Finally, the moderating effect of the type of publication is assessed as a supplement to the publication bias test.

## Methods

### Literature Search and Inclusion Criteria

Three searching strategies were used to find relevant studies. First, we searched online databases EBSCO, Elsevier Science Direct, PsycINFO, ProQuest, Springer, SAGE, Wiley, Summon, and Google Scholar, using a set of search terms including paternalistic leadership, benevolence leadership, morality leadership, authoritarian leadership, innovation, creativity, creative behavior, China, and Chinese to collect studies published in English. Studies published in Chinese were collected by searching Chinese online databases CNKI, Wanfang Data, VPCS, Taiwan Academic Literature Database, Superstar Discovery, and Baidu Scholar, using a set of Chinese search terms translated from the English search terms above. Second, we carried out ancestor searches according to the reference lists of review articles and reports we obtained. Third, we contacted some scholars in this area to find out if there was any unpublished work they had conducted. Databases were searched up to January 2022.

**Figure 1:**
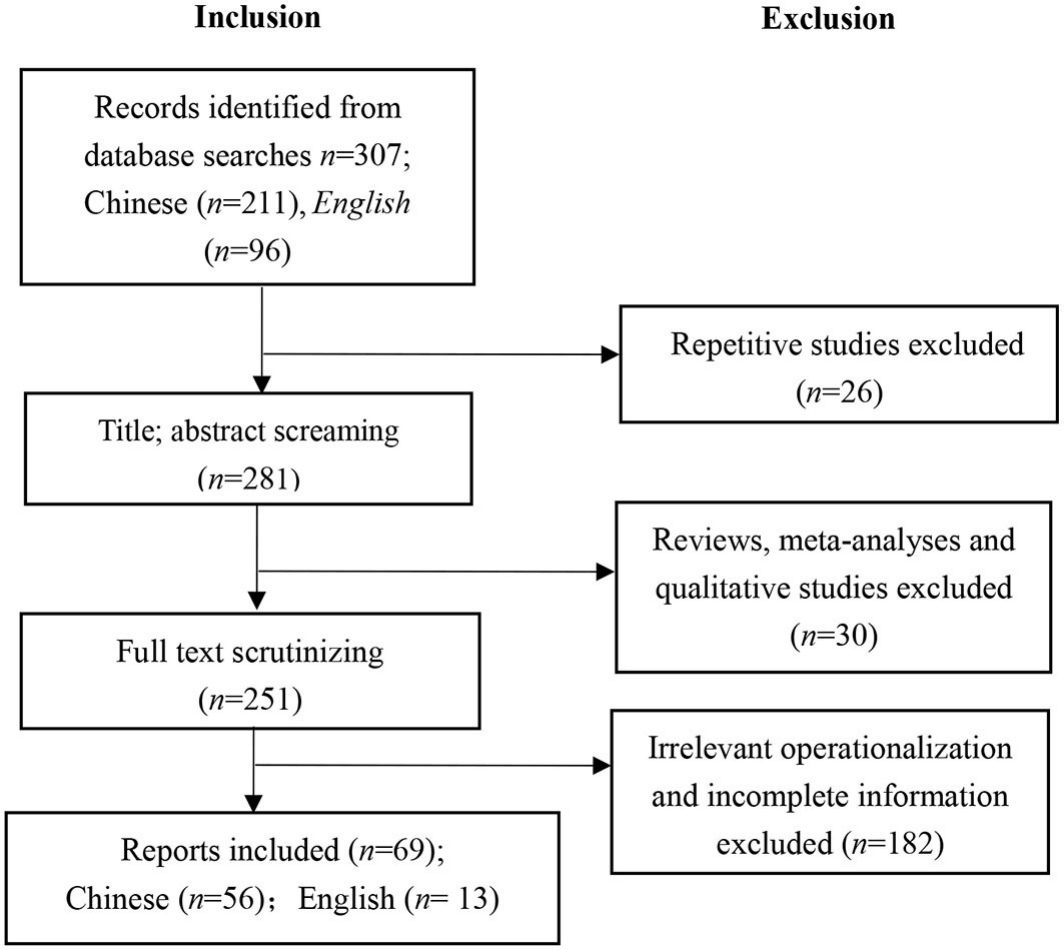
Literature search and inclusion diagram.

Studies included in this meta-analysis had to meet the following criteria: First, they had to be quantitative studies, and reviews and qualitative studies were excluded. Second, studies should report the measures. Third, studies had to adopt the measures of paternalistic leadership, which are relevant to the conceptualization proposed by Farh and Cheng (2000). Fourth, the information needed to calculate the overall effect sizes should be fully reported, including the sample size and *r*, or *t* value, *F*-value, or χ^2^ that can be converted into *r*. Fifth, the selected samples must be independent of each other. If multiple studies are retrieved from the same sample, only one of them would be included. The procedures for inclusion and exclusion are presented in Figure 1. Sixth, only studies among Chinese employees (including employees from mainland China, Hong Kong, Macao, and Taiwan) were included in the analysis.

Finally, 69 studies (13 English articles and 56 Chinese articles) were included in this study. The literature screening process is shown in Figure 1.

### Coding of Studies

For each study/independent sample, we coded (1) author and year of publication, (2) sample size, (3) effect size (*r*), (4) percent female respondents, (5) outcome measure, (6) type of publication (published and unpublished), (7) percent of employees with a college diploma or above, (8) type of data collection method (cross-sectional and longitudinal), and (9) type of evaluation method (supervisor- and self-evaluation). According to the coding standards proposed by Lipsey and Wilson (2001), Independent samples were used as the coding unit, and each independent sample was coded once. If there were multiple independent samples in a study, they were coded separately. The coding result is presented in Table 1. Sample characteristic is presented in Table 2.

**Table 1:** Sample information.

**References**	***n***	**Type of publication**	**% College**	**% Female**	**Type of evaluation**	**Data collection**	**Outcome measure**	***r***_**BL**_	***r***_**ML**_	***r***_**AL**_
Cai (2017)	172	Unpublished	97	52.3	Self	Cross-sectional	SB	0.201	0.256	−0.151
Cai et al. (2018)	568	Published	NA	46.5	Supervisor	Cross-sectional	Other			0.307
Chang et al. (2016)	637	Published	96.1	52	Self	Cross-sectional	Other	0.5	0.46	−0.24
Chen and Hou (2016)	291	Published	NA	19	Supervisor	Longitudinal	Other		0.11	
Chen et al. (2013)	176	Published	NA	13.7	Supervisor	Cross-sectional	ZG	0.56	0.42	−0.39
Chen (2018)	251	Unpublished	95.63	50.6	Self	Cross-sectional	SB	0.289	0.328	0.23
Chen et al. (2019)	448	Published	NA	44.5	Self	Cross-sectional	Other	0.31	0.412	−0.136
Cheng (2020)	282	Unpublished	92.2	56.7	Self	Cross-sectional	Cri	0.386	0.445	−0.359
Du and Wang (2020)	358	Published	NA	48.3	Self	Cross-sectional	SB	0.488	0.499	−0.384
Fang (2021)	224	Unpublished	NA	28.7	Supervisor	Cross-sectional	JA		0.34	
Feng (2009)	361	Unpublished	NA	45.2	Self	Cross-sectional	Other	−0.059	−0.038	0.088
Fu et al. (2012)	159	Published	NA	NA	Self	Cross-sectional	JA	0.31		−0.13
Gao (2013)	191	Unpublished	NA	NA	Supervisor	Cross-sectional	Other	0.19	0.03	−0.1
Ge (2012)	304	Unpublished	94.08	49.67	Self	Longitudinal	Other	0.37	0.41	−0.2
Gu et al. (2018)	325	Published	74.5	13.5	Supervisor	Cross-sectional	other			−0.23
Gu et al. (2020)	233	Published	91.42	31.33	Supervisor	Cross-sectional	ZG	0.18	0.19	−0.03
Gu et al. (2020)	125	Published	100	39.2	Supervisor	Cross-sectional	ZG	0.06	0.17	−0.01
Gu et al. (2015)	160	Published	93.12	28.12	Supervisor	Cross-sectional	ZG		0.33	
Guo et al. (2018)	192	Published	NA	56.2	Supervisor	Longitudinal	other			−0.2
Han (2018)	384	Published	95.6	45.7	Self	Cross-Section	Other	0.739	0.645	−0.415
Hou et al. (2019)	190	Published	NA	NA	Supervisor	Cross-sectional	Other	0.494	0.558	0.414
Huang (2012)	281	Unpublished	96.8	42.3	Supervisor	Longitudinal	JA	0.073	0.154	−0.15
Jia (2016)	193	Unpublished	95.83	46.11	Self	Cross-sectional	Other	0.59	0.43	−0.32
Jiang and Gu (2015)	167	Published	NA	31.7	Supervisor	Cross-sectional	ZG	0.38		
Jin et al. (2016)	127	Published	NA	NA	NA	Cross-sectional	Other	0.145	0.195	0.39
Li and Wu (2019)	2884	Published	89.28	52.74	Self	Cross-sectional	Other	0.452	0.37	0.134
Li and Wang (2021)	230	Published	63	43.3	Supervisor	Cross-sectional	JA	0.338	0.109	−0.316
Li et al. (2014)	312	Published	89.1	50	Self	Cross-sectional	SB	0.195	0.2	−0.126
Liang (2020)	325	Published	NA	NA	Self	Cross-sectional	SB	0.769	0.789	−0.732
Liu (2016)	436	Unpublished	100	38.4	Self	Cross-sectional	SB	0.163	0.067	−0.176
Liu (2018)	447	Unpublished	95.08	52.13	Self	Cross-sectional	Other	0.504	0.426	0.246
Ma (2012)	113	Unpublished	74	NA	Supervisor	Longitudinal	Other	0.22	0.306	−0.202
Ma and Zhang (2018)	232	Published	94.8	51.7	Supervisor	Longitudinal	JA			−0.321
Pan et al. (2013)	194	Published	NA	49	Supervisor	Cross-sectional	other			−0.01
She (2020)	290	Unpublished	NA	37.59	Self	Cross-sectional	Cri	0.223	−0.029	−0.184
Shen et al. (2017)	215	Published	70.3	54.4	Supervisor	Longitudinal	SB	0.31		
Shi and Li (2014)	510	Published	NA	NA	Self	Cross-sectional	other	0.626		−0.295
Tang (2016)	181	Unpublished	90.06	56.91	Self	Cross-sectional	Other	0.231	0.241	−0.072
Tian and Sanchez (2017)	302	Unpublished	93	44	Supervisor	Cross-sectional	SB	0.37		−0.02
Wang and Cai (2016)	1123	Published	74.8	NA	Self	Cross-sectional	Other	0.326	0.414	−0.082
Wang and Cheng (2010)	167	Published	NA	37	Supervisor	Cross-sectional	ZG	0.33		
Wang and Liu (2017)	447	Published	NA	NA	Self	Cross-sectional	Other	0.403	0.38	−0.246
Wang and Xing (2019)	233	Published	31.2	19.3	Self	Longitudinal	other			0.041
Wang (2019)	310	Published	NA	NA	Self	Cross-sectional	Cri	0.407	−0.355	−0.028
Wang et al. (2019)	378	Published	NA	58.2	Self	Cross-sectional	Other	0.23	0.18	−0.08
Wang Z. et al. (2021)	441	Published	NA	55.1	Supervisor	Longitudinal	SB	0.35		
Wang (2015)	450	Published	NA	40.78	Supervisor	Cross-sectional	SB	0.431		−0.109
Wang (2018)	356	Unpublished	NA	NA	Self	Longitudinal	SB	0.718		−0.632
Wang Y. W. et al. (2021)	284	Published	NA	NA	Self	Cross-sectional	SB			0.207
Wang A. C. et al. (2018)	275	Published	NA	43.3	Supervisor	Cross-sectional	Other		0.37	
Wang and Wang (2019)	376	Published	NA	59	Self	Cross-sectional	SB	0.3		
Wei and Li (2021)	330	Published	NA	51.8	Self	Cross-sectional	other	0.68		
Wei and Wang (2020)	230	Published	NA	41.3	Supervisor	Cross-sectional	JA		0.45	
Wei et al. (2018)	250	Published	NA	32.2	Self	Cross-sectional	other	0.426		
Wei et al. (2017)	325	Published	74.2	13.5	Self	Cross-sectional	ZG		0.161	
Wu (2018)	196	Published	99.99	45.92	Self	Cross-Section	Other	0.465	0.502	−0.302
Xia (2020)	1305	Published	NA	35.63	Supervisor	Longitudinal	other	0.25		
Xia et al. (2021)	297	Published	100	NA	Supervisor	Longitudinal	other	0.4		0.3
Xie (2019)	357	Published	NA	NA	Self	Cross-sectional	SB	0.258		
Xu et al. (2014)	208	Published	93.3	33.2	Supervisor	Cross-sectional	ZG		0.213	
Xu (2020)	358	Unpublished	100	47.6	Self	Cross-sectional	Other	0.441	0.394	−0.329
You (2007)	315	Unpublished	71.7	39.7	Supervisor	Cross-sectional	SB			−0.24
You (2020)	178	Unpublished	86.3	58.8	Self	Longitudinal	Other	0.315	0.26	0.114
Zeng (2012)	271	Unpublished	95.57	45	Self	Cross-sectional	Other	0.356	0.332	−0.128
Zeng (2020)	335	Published	96.4	44.8	Self	Cross-sectional	other			−0.559
Zhang (2016)	264	Unpublished	94.7	47	Self	Cross-sectional	Other	0.737	0.709	−0.605
Zhang et al. (2015)	301	Published	NA	NA	Self	Cross-sectional	Other	0.355	0.169	−0.092
Zhao and Nie (2018)	394	Published	100	48.22	Self	Cross-sectional	JA	0.74	0.61	−0.24
Zhou (2021)	522	Unpublished	100	49.8	Self	Cross-sectional	ZG	0.477	0.425	−0.4
Zhu (2009)	301	Unpublished	88.7	58.5	Self	Cross-sectional	JA	0.2685		−0.029

*69 studies, 13 studies in English, 56 studies in Chinese; SB, the innovative behavior scale developed by Scott and Bruce (1994); JA, the innovative behavior scale developed by Janssen (2000); ZG, the creativity scale developed by Zhou and George (2001); Cri, the Bootlegging innovation scale developed by Criscuolo et al. (2014); Other, other scales used twice or less by the studies included*.

**Table 2:** Sample characteristic.

**Characteristic**	**BL**		**ML**		**AL**	
	***k***	***n***	***k***	***n***	***k***	***n***
Outcome measure						
CRI	3	882	3	882	3	882
JA	5	1,365	5	1,359	6	1,597
SB	13	4,351	6	1,854	12	4,002
ZG	5	1,265	7	1,749	4	1,056
Other	25	12,175	24	10,542	30	12,630
Year of publication						
2009–2014	11	3,033	8	2,104	12	3,375
2015–2021	43	17,623	37	14,282	43	16,792
Type of publication						
Published	33	14,602	19	5,319	33	13,798
Unpublished	21	6,054	25	10,942	22	6,369
Type of evaluation						
Supervisor	16	4,883	14	2,627	17	4,530
Self	37	15,646	30	13,332	37	15,510
Data collection						
Cross-sectional	45	17,166	39	15,092	45	17,540
Longitudinal	9	3,490	5	1,167	10	2,627
% Female						
*Rang*e	13.7–59%	13.5–58.8%		13.5–58.8%	
% College						
*Range*	63–100%	63–100%		31.2–100%	
Overall	54	20,656	45	16,386	55	20,167

*BL, benevolent leadership; ML, moral leadership; AL, authoritarian leadership*.

### Data Analysis

#### Effect Size

Data were analyzed using Comprehensive Meta-Analysis software (CMA) 3.0. CMA uses Hedges–Olkin method (Hedges and Olkin, 1985; Borenstein et al., 2011) to transform and aggregate the correlation coefficients. This study used correlation coefficients to summarize the relationships between the three dimensions of paternalistic leadership and employees' innovation. Correlations were first transformed to Fisher's z to stabilize the variance. **Z**_i_ = **0.5**ln[(**1−r_i_**)/(**1+r_i_**)]. The *z*-value was then weighted and transformed back to *r*, the overall effect sizes. **r** = (**e**^**2z**^**−1**)/(**e**^**2z**^**+1**).

#### Model Selection

To examine whether the random-effects model or fixed-effect model should be selected to obtain the overall effect size, we used Cochran's *Q* statistic and *I*^2^ statistic as two indicators of the heterogeneity test. *Q* > critical value and *p* < 0.05 indicate that samples are heterogeneous, and a random-effects model is more recommended; otherwise, a fixed-effect model should be performed (Borenstein et al., 2010). *I*^2^ exceeding 25, 50, and 75%, respectively, indicates that low, medium, or high heterogeneity exists among the study samples (Higgins et al., 2003).

#### Publication Bias Test

Funnel plot and fail-safe number (*N*_fs_) were used to test the publication bias. *N*_*fs*_ coefficient is the number of studies that reported results required to refuse a conclusion. The larger *N*_fs_ is, the more reliable the meta-analysis results are. When *N*_*fs*_ is >5*k* +10, there is less possibility of publication bias (Rothstein et al., 2005).

#### Moderator Test

Mixed-effects between-level *Q* moderator analyses (Borenstein et al., 2010) were adopted to examine the moderating effects of categorical moderators, including outcome measure, the type of evaluation (self-and supervisor-evaluation), data collection (cross-sectional and longitudinal), the type of publication (published articles and unpublished theses and dissertations). Fixed-effect meta-regression (Borenstein et al., 2010) was used to examine the moderating effects of continuous moderators, including gender (the percentage of females), year of publication, and educational level (the percentage of employees with a college diploma or above).

## Results

### Sample Description

#### Heterogeneity

The results of heterogeneity are shown in Table 3. Cochran's *Q* statistics of studies on benevolent leadership (BL), moral leadership (ML), and authoritarian leadership (AL) reached a statistically significant level (*p* < 0.001). The *I*^2^ values of the three leadership styles and employee innovation were >75%. Therefore, it is more reasonable to fit random-effects models to compute the overall effect sizes in this study.

**Table 3:** Main effects and publication bias tests.

				**95% CI for** ***r***					
	***k***	***N***	***r***	***LL***	***UL***	***Z***	***Q***	***I^2^***	***N_fs_***
BL	54	20,656	0.396	0.344	0.445	13.627^***^	984.269^***^	94.615	46,234
ML	45	16,386	0.329	0.266	0.390	9.645^***^	857.072^***^	94.866	20,577
AL	55	20,167	−0.151	−0.220	−0.080	−4.158^***^	1399.605^***^	96.142	5,481

BL, benevolent leadership; ML, Moral leadership; AL, authoritarian leadership; k, the number of independent samples; N, cumulative number of samples; CI, confidence interval; LL, lower limit; UL, upper limit, Q value and its significance represent the degree of heterogeneity, and I^2^ represents the proportion of heterogeneity in the total variation; *p < 0.05, **p < 0.01,

*** *p < 0.001*.

### Publication Bias

**Figure 2:**
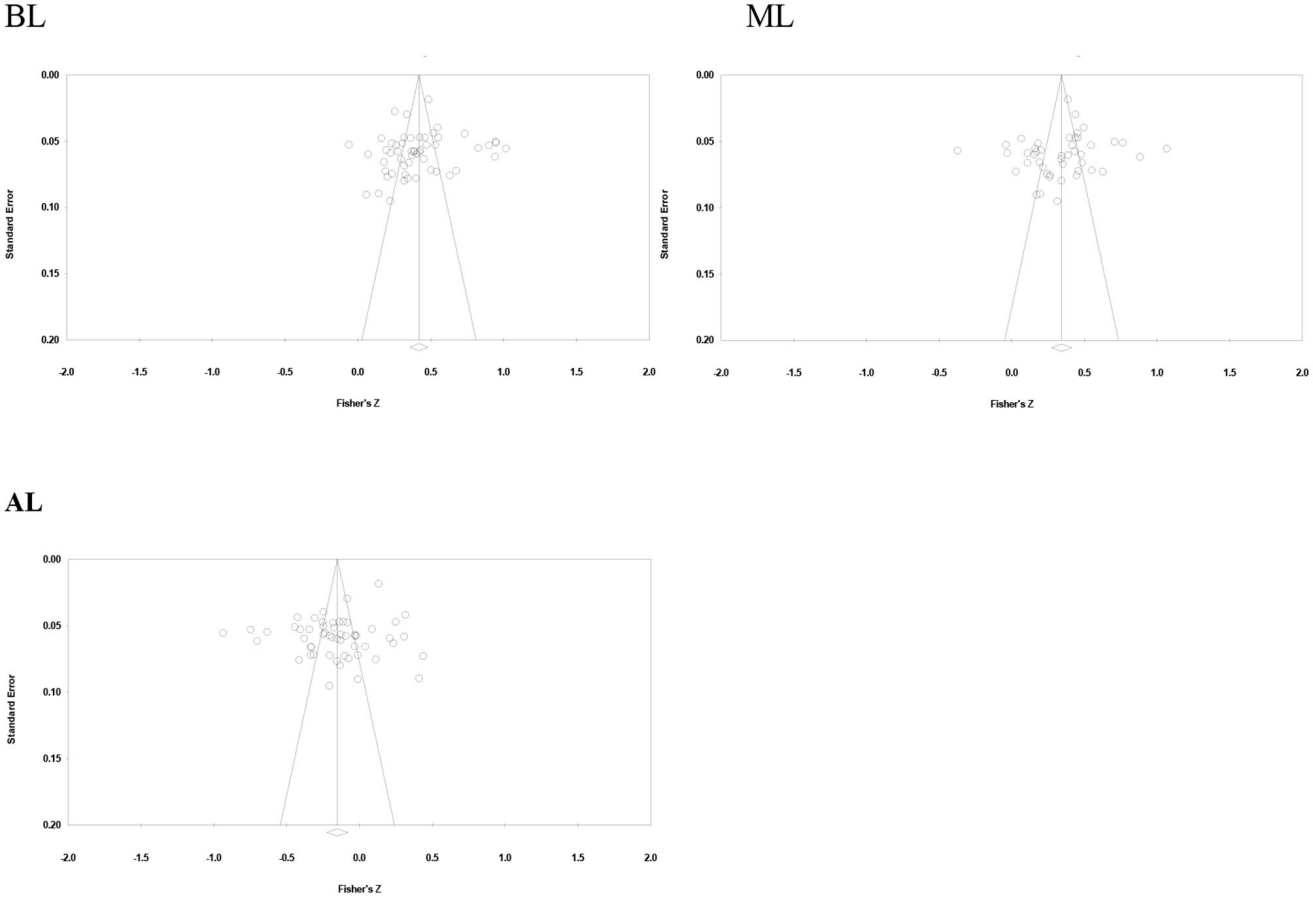
Funnel plot; BL, benevolent leadership; ML, moral leadership; AL, authoritarian leadership.

The results of publication bias tests are shown in Figure 2 and Table 3. According to the funnel plots of studies, most studies were located at the top and evenly distributed on both sides, and the funnel plots are generally symmetrical.

In addition, according to Table 3, the *N*_fs_ coefficients of BL, ML, and AL are 46,234, 20,577, and 5,481, respectively, which are much higher than 5*k* +10, indicating that this study is not affected by publication bias and the research conclusion is robust and reliable.

### Main Effect Analysis

Table 3 shows the results of the main effect tests. BL (*r* = 0.40, *p* < 0.001) and ML (*r* = 0.33, *p* < 0.001) were significantly positively correlated with employees' innovation with medium effect sizes, while AL was significantly negatively correlated with employees' innovation with small effect size (*r* = −0.15, *p* < 0.001), supporting H1, H2, and H3.

### Moderator Analysis

For continuous moderators, the results of meta-regression are shown in Table 4. The moderating effects of gender (the percentage of female respondents) were significant. The percentage of females in the samples could positively predict the effect sizes of the relationships between the three dimensions of paternalistic leadership and innovation, indicating that the more female employees in the samples, the stronger the positive effects of BL and ML, and the smaller the negative effect of AL on innovation, supporting H4.

**Table 4:** Moderating effects of continuous variables (meta-regression analysis).

**Moderator**		***k***	***Estimate***	***SE***	***LL***	***UL***	***Z***	***Q _model_***
BL								
	% Female	38	0.003	0.001	0.001	0.005	3.222^**^	10.381^***^
	Year of publication	54	0.017	0.002	0.012	0.021	6.933^***^	48.063^***^
	% College	29	0.006	0.001	0.004	0.008	5.696^***^	32.450^***^
ML								
	% Female	35	0.006	0.001	0.004	0.008	6.724^***^	45.218^***^
	Year of publication	44	0.021	0.003	0.015	0.027	7.093^***^	50.305^***^
	% College	28	0.005	0.001	0.002	0.007	4.136^***^	17.104^***^
AL								
	% Female	39	0.002	0.001	0.000	0.004	1.731	2.996
	Year of publication	55	0.001	0.002	−0.003	0.006	0.617	0.381
	% College	33	−0.003	0.001	−0.004	−0.001	−3.889^***^	15.127^***^

BL, benevolent leadership; ML, moral leadership; AL, authoritarian leadership; LL, lower limit; UL, upper limit; *p < 0.05,

** *p < 0.01*,

*** *p < 0.001*.

The education level of employees significantly moderated the relationship between paternalistic leadership and employees' innovation. The percentage of employees with a college diploma or above could positively predict the effect sizes of BL and ML but negatively predict the effect sizes of AL, supporting H5 and H6.

Year of publication could positively and significantly moderate the relationship between BL, ML, and employees' innovation. The positive effects of BL and ML on innovation in recent years were greater than those about a decade ago. However, the year of publication could not moderate the relationship between AL and innovation, which does not support H10.

For categorical moderators, the results of between-level *Q* moderator analyses are shown in Table 5. The type of evaluation (supervisor- vs. self-evaluation) could moderate the relationships between BL, ML, and innovation significantly (*p*s ≤ 0.063), and there were stronger correlations under the self-evaluation of innovation, while the moderating effect was not significant for AL. The moderating effects of outcome measure, data collection method, and publication type were not significant.

**Table 5:** Moderating effects of categorical variables (subgroup analysis).

	**Moderator**	**k**	**n**	**r**	**LL**	**UL**	**Z**	**Qb**
**BL**	**Outcome measure**							1.220
	Cri	3	882	0.341	0.225	0.448	5.501^***^	
	JA	5	1365	0.376	0.063	0.621	2.335^*^	
	SB	13	4351	0.395	0.268	0.509	5.716^***^	
	ZG	5	1265	0.394	0.260	0.513	5.432^***^	
	Other	25	12175	0.414	0.342	0.482	10.195^***^	
	**Type of evaluation**							6.709^**^
	supervisor	16	4883	0.317	0.253	0.377	9.311^***^	
	Self	37	15646	0.433	0.369	0.493	11.932^***^	
	**Data collection**							0.558
	Cross-sectional	45	17166	0.405	0.348	0.458	12.743^***^	
	Longitudinal	9	3490	0.351	0.212	0.475	4.766^***^	
	**Type of publication**							1.299
	Published	33	14602	0.421	0.359	0.478	12.110^***^	
	Unpublished	21	6054	0.356	0.257	0.447	6.659^***^	
**ML**	**Outcome measure**							3.749
	Cri	3	882	0.026	−0.428	0.470	0.105	
	JA	5	1359	0.349	0.129	0.535	3.051^**^	
	SB	6	1854	0.393	0.112	0.616	2.687^**^	
	ZG	7	1749	0.279	0.178	0.374	5.279^***^	
	Other	24	10542	0.359	0.294	0.422	10.030^***^	
	**Type of evaluation**							2.073
	supervisor	14	2627	0.274	0.189	0.355	6.148^***^	
	Self	30	13332	0.357	0.277	0.432	8.245^***^	
	**Data collection**							1.679
	Cross-sectional	40	15092	0.339	0.271	0.403	9.200^***^	
	Longitudinal	5	1167	0.249	0.125	0.365	3.871^***^	
	**Type of publication**							0.582
	Published	19	5319	0.301	0.206	0.391	5.961^***^	
	Unpublished	26	10942	0.349	0.264	0.429	7.552^***^	
**AL**	**Outcome measure**							2.779
	Cri	3	882	−0.194	−0.374	0.001	−1.947^0.052^	
	JA	6	1597	−0.200	−0.290	−0.105	−4.105^***^	
	SB	12	4002	−0.210	−0.382	−0.023	−2.200^*^	
	ZG	4	1056	−0.220	−0.423	0.004	−1.925^0.054^	
	Other	30	12630	−0.103	−0.196	−0.009	−2.157^*^	
	**Type of evaluation**							3.450^0.063^
	supervisor	17	4530	−0.067	−0.180	0.048	−1.142	
	Self	37	15510	−0.202	−0.285	−0.116	−4.547^***^	
	**Data collection**							0.005
	Cross-sectional	45	17540	−0.152	−0.228	−0.075	−3.841^***^	
	Longitudinal	10	2627	−0.145	−0.325	0.045	−1.494	
	**Type of publication**							0.332
	Published	33	13798	−0.134	−0.224	−0.042	−2.840^***^	
	Unpublished	22	6369	−0.176	−0.282	−0.066	−3.122^**^	

*BL, benevolent leadership; ML, moral leadership; AL, authoritarian leadership; superviso r, supervisor-evaluation; self, self-evaluation; LL, lower limit, UL, upper limit*,

* *p < 0.05*,

** *p < 0.01*,

*** *p <0.001*.

## Discussion

### Relationship Between Paternalistic Leadership and Employee Innovation

This meta-analysis demonstrates medium positive associations of benevolent leadership (BL) and moral leadership (ML) with Chinese employees' innovation and a small negative association between authoritarian leadership (AL) and Chinese employees' innovation.

The positive effects of BL and ML and the negative effect of AL found in this study are similar to some previous empirical studies (e.g., Wu, 2018; Cheng, 2020) and a last multinational meta-analysis focusing on employee overall performance (Hiller et al., 2019). Our findings verified the robustness of the positive correlation between BL and ML and employee innovation and the negative correlation between AL and employee innovation among Chinese employees. These findings are consistent with our H1 and H2 proposed based on the employee creativity formation mechanism model (Wang et al., 2010) and self-determination theory (Deci and Ryan, 1985, 2000). That said, effect sizes differed depending on several moderators, which we now discuss.

### Moderating Factors

The associations between the three aspects of paternalistic leadership and employee innovation are moderated by the percentage of females in the samples, the percentage of employees with a higher educational level, publication year, and evaluation type.

Gender is one of the essential moderators. A higher proportion of female employees in an organization will result in stronger positive associations of BL and ML with employees' innovation. Meanwhile, it will also result in a weaker negative relationship between authoritarian leadership and employees' innovation. This result verifies our hypothesis and supports role congruity theory, indicating that women's relatively greater relationship orientation (Boatwright and Forrest, 2000) might not only be a promotive factor but a protective factor in the relationship between paternalistic leadership and employee innovation. To date, all the research performed have been focusing on the role of women in the workplace (e.g., Browne, 1998; Cheng et al., 2011; Kato and Kodama, 2017; Zhou and Zhou, 2017; Sposato, 2021). Future research can further examine the unique role of female employees in innovation.

Employee educational background also plays a role in the association between paternalistic leadership and innovation. A higher proportion of employees with a college diploma or above in samples can strengthen the positive associations of BL and ML with employee innovation and the negative association between AL and innovation. Such findings are consistent with our H5 and H6 proposed based on the previous understanding of what educated employees value at work (respect, emotional incentive, and autonomy; Oldham and Cummings, 1996; Shalley et al., 2004).

Publication year moderates the associations of BL and ML with innovation, but it cannot moderate the relationship between AL and innovation. With the development of Chinese society in the past 10 years, BL and ML are thus becoming increasingly conducive to innovation. This finding is consistent with H9. Current Chinese employees value respect, emotional incentive, and justice at work more than before, probably caused by the ongoing socioeconomic and cultural changes in China (see Xu and Hamamura, 2014; Cao, 2020). Thus, BL and ML initially become greater promotors for their intrinsic motivation, resulting in more innovative behaviors. However, unexpectedly, the association between AL and innovation cannot be moderated by publication year, which is inconsistent with our hypothesis about the changing attitude of Chinese employees toward authoritarian leaders. A possible explanation is that although Chinese employees value a respectful, caring, and fair working environment in recent years more than before, their attitude toward authority in the workplace has not changed essentially. Authority has been long deeply rooted in Chinese Confucian culture and Chinese people's minds. It has also played a significant role in every corner of society and people's lives. Thus, people's attitudes toward authority tend to be stable.

The significant/marginally significant moderating effects of evaluation types of innovation in the associations of BL and AL with innovation are partially consistent with our H7 and H8, indicating that common method biases might have existed in previous studies. As predicted, the mean effect size in past studies that adopted self-evaluation (*r* = 0.433) is larger than those that used supervisor evaluation (*r* = 0.317) in the relationship between BL and innovation. The relatively small effect size of 0.317 is still statistically significant and considered medium. That is, although a single study may overestimate the effect size because of the common method bias caused by self-evaluation, the impact of self-evaluation is not that essential in general and is relatively acceptable for this meta-analysis. However, in the association between AL and innovation, the difference between self and supervisor evaluation is essential, and supervisor evaluation yields a weak mean effect size (−0.067 vs. −0.202) and is insignificant. This finding implies that the association between AL and innovation can be overestimated. Thus, we should be cautious when explaining related results. The moderating effect of evaluation type is insignificant in the relationship between ML and innovation. No common method bias, which impacts the association between ML and innovation, is found.

The moderating effects of the type of outcome measure (innovation scales that the studies used) were not significant. The associations are stable over different measurement tools in general except for the weak association between ML and innovation measured by Cri (Criscuolo's innovation scale). This might be because only three studies are adopting Cri, and the result is more easily influenced by the large between-study variance caused by random errors.

Finally, the type of publication and the type of data collection method are not moderators between paternalistic leadership and innovation. These results are inconsistent with our hypotheses, indicating that the results of this meta-analysis are not influenced by publication bias caused by previously published studies or common method bias caused by previous cross-sectional studies.

### Limitations and Future Research

The present study still has several limitations: First, the vast majority of the samples included in this meta-analysis are from mainland China, and only two Taiwanese samples are included. We did not compare the potential difference between samples from the mainland and Taiwan because of the highly uneven sample numbers. Future research can compare the results from different regions of China, especially from Taiwan, Hong Kong, or Macao (when there are more empirical studies from these regions), which are quite different from the mainland in terms of economy, societal values, and culture.

Second, as most existing meta-analyses focusing on paternalistic leadership did, we treated paternalistic leadership as three separate dimensions without testing their interactions. However, in real workplace settings, the three aspects of paternalistic leadership usually appear together. In future research, some more advanced meta-analysis techniques, for example, meta-analytic criterion profile analysis (MACPA), are supposed to be adopted to comprehensively analyze the interactions of the three dimensions of paternalistic leadership.

Third, the moderators located in this research are still limited. A future study can explore the moderating effects of other moderators once there are sufficient information reported, especially team-level factors, for example, the characteristics of leaders (e.g., gender, educational level, and professional background), and organizational level factors such as industries, company size, and the type of company (state-owned, private, or public).

In addition, a future study can further elaborate on the associations between paternalistic leadership and different types of innovation (e.g., bootleg innovation, disruptive innovation, architectural innovation, radical innovation, etc.) and examine the difference among the different innovation types to find out which kind of innovation paternalistic leadership is most beneficial or harmful to.

### Theoretical Implications

First, as mentioned above, our findings on the association between the three aspects of paternalistic leadership and innovation among Chinese employees are consistent with the hypotheses based on the employee creativity formation mechanism model and SDT, demonstrating the applicability of these two theories in the Chinese organizational context.

Second, the result of the moderator test on gender verified role congruity theory among Chinese employees. Our findings also reveal female employees' promotive-protective role in innovation. Future research can further construct a more holistic model for females' promotive-protective role in innovation in workplace settings.

Third, our findings on the moderating role of publication year partially support the theory of value change (Inkeles, 1969; Inglehart, 1997). However, publication year could not moderate the relationship between AL and innovation, indicating employees' stable attitude toward authoritarianism. As our analysis above, it might be because authoritarianism is a relatively stable component in Chinese culture. This finding reminds researchers that they should pay attention to the differences in their cultural backgrounds when using the theory of value change.

### Practical Implications

This study found that benevolent leadership and moral leadership are beneficial for employee innovation, and authoritarian leadership might be harmful to innovation. Furthermore, the associations are moderated by subordinates' educational level and gender. According to these findings, we develop several practical implications of our findings for personnel appointments, organizational policy, and team leaders.

#### For Personnel Appointments

Leaders with greater BL and ML attributes than AL attributes should probably be considered to be in charge of a team or project with high requirements for innovation (e.g., R&D or marketing). And they might also fit better with a team composed of educated members. In addition, because a higher proportion of female members may be a promotive and protective factor for innovation, those creating an innovation project team may consider recruiting more women as team members.

#### For Organizational Policies

To facilitate employee innovation, organizations can encourage leaders to practice BL and ML through incentives, while policies that mitigate the effects of leaders' authority should also be implemented. For example, employees should be given the opportunity and channel to complain when they are subjected to improper authoritarian treatment by their superiors.

#### For Team Leaders

In future management practice, paternalistic leaders may improve their awareness of their leadership styles, emphasize authority less, and focus more on other aspects of paternalism (benevolence, morality, and responsivity) to foster employee innovation, especially when working with educated subordinates. In addition, leaders may also pay attention to the gender difference of subordinates in the effects of authoritarianism which may cause less innovative behaviors or more negative outcomes among male workers than female workers. Therefore, to mitigate the influence of AL on innovation, paternalistic leaders may show less authoritarianism, particularly to male subordinates.

Furthermore, in today's management practice, the function or dysfunction of paternalism might depend on whether a paternalistic leader can keep pace with the times. Currently, leaders are required to be flexible and agile due to the uncertain and rapidly changing circumstances that a team or organization may constantly encounter (Chen et al., 2022). Although paternalism is an order leadership style, it is not necessarily the opposite of agility. On the contrary, paternalistic leadership may function better in combination with agile leadership, the capability to adapt, renew itself, and thrive in a rapidly ambiguous, changing, and raging environment (Vecchiato, 2015; Salmen and Festing, 2021). Facing different objects and situations, different combinations or patterns of the three dimensions of paternalistic leadership can bring different leadership effects (Niu et al., 2009; Wang, 2018). Our findings also indicate that different aspects of paternalism affect innovation differently. Therefore, in managerial practice, paternalistic leaders can flexibly adjust their weight on each aspect of paternalistic leadership (benevolence, morality, and authority) and find the best combination of the three dimensions for the situations.

## Data Availability Statement

The datasets presented in this study can be found in online repositories. The names of the repository/repositories and accession number(s) can be found in the article/supplementary material.

## Author Contributions

LL and KZ contributed to the study's conception and design, performed data analysis, and wrote the first draft of the manuscript. Material preparation and data collection were performed by SZ and YW. All authors commented on previous versions of the manuscript, read, and approved the final manuscript.

## Funding

This work was supported by the Social Science Research Plan of Guangan 2020 (No. 2020-64) and the Political Science Research Funds of Xiao Ping Executive Leadership Academy, China.

## Conflict of Interest

The authors declare that the research was conducted in the absence of any commercial or financial relationships that could be construed as a potential conflict of interest.

## Publisher's Note

All claims expressed in this article are solely those of the authors and do not necessarily represent those of their affiliated organizations, or those of the publisher, the editors and the reviewers. Any product that may be evaluated in this article, or claim that may be made by its manufacturer, is not guaranteed or endorsed by the publisher.
